# A comparative study of cold- and warm-adapted Endonucleases A using sequence analyses and molecular dynamics simulations

**DOI:** 10.1371/journal.pone.0169586

**Published:** 2017-02-13

**Authors:** Davide Michetti, Bjørn Olav Brandsdal, Davide Bon, Geir Villy Isaksen, Matteo Tiberti, Elena Papaleo

**Affiliations:** 1 The Centre for Theoretical and Computational Chemistry, Department of Chemistry, Faculty of Science and Technology, The Arctic University of Norway, Tromsø, Norway; 2 School of Biological and Chemical Sciences, Queen Mary University of London, Mile End Road, London E1 4NS, United Kingdom; 3 Computational Biology Laboratory, Danish Cancer Society Research Centre, Strandboulevarden 49, Copenhagen, Denmark; 4 Department of Biotechnology and Biosciences, University of Milano-Bicocca, P.zza della Scienza 2, Milan, Italy; Università degli Studi di Milano, ITALY

## Abstract

The psychrophilic and mesophilic endonucleases A (EndA) from *Aliivibrio salmonicida* (VsEndA) and *Vibrio cholera* (VcEndA) have been studied experimentally in terms of the biophysical properties related to thermal adaptation. The analyses of their static X-ray structures was no sufficient to rationalize the determinants of their adaptive traits at the molecular level. Thus, we used Molecular Dynamics (MD) simulations to compare the two proteins and unveil their structural and dynamical differences. Our simulations did not show a substantial increase in flexibility in the cold-adapted variant on the nanosecond time scale. The only exception is a more rigid C-terminal region in VcEndA, which is ascribable to a cluster of electrostatic interactions and hydrogen bonds, as also supported by MD simulations of the VsEndA mutant variant where the cluster of interactions was introduced. Moreover, we identified three additional amino acidic substitutions through multiple sequence alignment and the analyses of MD-based protein structure networks. In particular, T120V occurs in the proximity of the catalytic residue H80 and alters the interaction with the residue Y43, which belongs to the second coordination sphere of the Mg^2+^ ion. This makes T120V an amenable candidate for future experimental mutagenesis.

## Introduction

Enzymes isolated from organisms living in cold environments (psychrophiles, -20 to 15°C) are interesting targets for both industrial applications and fundamental research of protein folding and catalysis [1–4]. Temperature is normally regarded as one of the major external factors affecting the adaptive capacities of life, influencing molecular motions and chemical reaction rates as well as the physical properties of water. A key issue with lowering the temperature is the exponential decrease in the enzyme catalytic rates. Despite this restraint cold-adapted enzymes display higher catalytic activity at low to moderate temperatures when compared to their warm-active counterparts. Along with an increased k_cat_, the majority of psychrophilic enzymes also display a higher K_M_ [5,6]. Understanding how cold-active enzymes avoid the decrease in their activity as the temperature is lowered is the key problem of enzymatic temperature adaptation. The main strategy to compensate for the reduced temperature and its effect on the catalytic reaction rates is lowering of ΔH^‡^, making the reactions less temperature-dependent. However, this is also accompanied by an unfavorable activation entropy (TΔS^‡^), thereby dampening the effect of reducing the enthalpy [7,8]. To date, all cold-active enzymes catalyze their reaction with a lower activation enthalpy and more negative activation entropy compared to their mesophilic counterparts, thus representing the fingerprint for cold-active enzymes [5,6,9].

An increased flexibility of the cold-active enzymes, either localized in the proximity of the active site or in more distant parts of the structure has been suggested as one of the determinant for enzyme cold adaptation [10–18]. This flexibility has also been linked to the observed decrease in the structural thermostability of cold-active enzymes [14,19]. Whether the thermolability is a consequence of higher flexibility or of the lack of evolutionary pressure on stability is still unclear and changes in overall flexibility were not observed in all the cold-adapted enzymes [20,21].

The properties required for enzymes to function at low temperatures can be achieved in many different ways by amino acids substitutions in key protein regions, and the structural determinants can also vary among different families of enzymes [13,20,21]. In general cold-adapted enzymes increase their flexibility by destabilization of their structure, either locally or globally, through a decrease in the different class of stabilizing interactions: salt-bridges, hydrogen bonds and aromatic interactions. A lower number of disulfide bridges, a more hydrophobic/hydrophilic or negatively charged exposed surface and a less compact hydrophobic core have been found to play a role in cold adaptation [5]. Regarding the preference of amino acids in psychrophilic sequences, comparative studies have shown a higher number of glycines, in particular in loop regions, and a lower number of prolines and arginines [20]. Longer surface loops have been also linked to higher flexibility in cold-adapted enzymes [22,23].

Here we used the psychrophilic/halophilic and mesophilic/halotolerant endonuclease A from *Aliibrio salmonicida* (VsEndA) and *Vibrio cholerae* (VcEndA) as model systems to study structural properties related to cold adaptation. These systems were chosen due to the availability of experimental data [24–27], their high degree of sequence identity (71%) and structural similarity with a backbone root mean square deviation (RMSD) of approximately 0.087 nm [24]. Endonuclease A is a periplasmic/extracellular enzyme known to cleave DNA and RNA at unspecific internal sites. It relies on a histidine residue (H80) as a general base to activate a water molecule, which in turn acts as a nucleophile for an in-line attack of the scissile phosphate [28,29]. The magnesium (Mg^2+^) ion, located in the ββ-α motif, is involved in the binding of the scissile phosphate, in applying a strain over the DNA molecule that is released upon product formation and in the stabilization of the charged transition state PO_5_^2-^. Endonucleases A are monomers of approximately 25 kDa (~210 aminoacids) and belong to a family of non-specific metal-dependent endonucleases, which share a ββ-α motif in the catalytic site (S1 Fig) [30]. The overall 3D structure is formed by eleven α-helices and seven β-strands (S1 Fig). A conserved sequence motif EWEH includes the metal binding residue E79 and the catalytic residue H80. The N127 metal-coordinating residue is also located in the structural ββ-α motif on the α7 helix (S1 Fig). The active site is formed by a large cleft surrounded by α1 and β1–2 on top, α2 and α3–5 on the side and β5–6 at the bottom (S1 Fig). β3 and α7 are located in the center, making contact with DNA along the minor groove.

Here, we report the analysis of a multiple sequence alignment, all-atom MD simulations and protein structure networks (PSN). The combination of Root Mean Square Fluctuation (RMSF) analysis, PSN and the multiple sequence alignment proved to be a valuable tool to overcome the weakness inherent in the analyses of static structures. The multiple sequence alignment allowed to discriminate between amino acidic substitutions that are more likely to be related to cold adaptation from those that are conserved in the mesophilic orthologues or due to a genetic drift. Three amino acidic substitutions emerged from our study: T120V, I141S and A166S, also supported by the atom-level analyses provided by RMSF and PSN-MD calculations (in the notation employed henceforth for amino acidic substitutions the first amino acid belongs to VcEndA and the second to VsEndA).

## Materials and methods

The sequence numbering employed is referred to the *Vibrio vulnificus* endonuclease (Vvn) PDB structure (1OUP)[28].

### Multiple sequence alignment

We collected the homologous sequences of VsEndA through a Blastp search [31] in the *non-redundant protein sequences* database. In particular, we used the Blosum 62 substitution matrix [32] with gap cost of 11 for opening and 1 for extension and a word size of 6. We retained only sequences with at least 50% of sequence identity with the query for further analyses. We trimmed the sequence dataset to reduce redundancy and to remove signatures related to different adaptation traits, retaining only 54 different protein sequences. More in details, we discarded all the mesophilic non-halophilic organisms to ensure that only one type of environmental adaptation was considered (i.e., temperature). Secondly, we included only mesophilic variants of the *Vibrio* genus with optimum temperature around 37°C. Thirdly, when a set of sequences featured comparable sequence identity with respect to VsEndA, only one sequence was retained in the dataset to remove redundancy. Finally, we discarded all the sequences with unclear temperature optima. The multiple sequence alignment was carried out with Clustal Omega [33] using default parameters. The visualization and rendering of the multiple sequence alignment was carried out with ESPript 3 [34].

### Molecular dynamics simulations

We prepared the initial structures for MD simulations starting from the X-ray structures of VsEndA (PDB entry: 2PU3, 1.5 Å Resolution [24]) and VcEndA (PDB entry: 2G7F, 1.95 Å Resolution [26]) using the Protein Preparation wizard from Maestro package [35]. In the preparation, we retained the crystal water molecules and we removed the chloride ion from the 2PU3 entry due to negligible contacts with the protein. We also carried out two MD simulations of a VsEndA mutant variant where we introduced a salt-bridge network observed in the C-terminal region of the mesophilic enzyme. In particular, we replaced the residues, K226, N179 and Q222 with arginine and glutamate, respectively (N179E,Q222R, K226E). We used Pymol to model the mutations [36].

We carried out the MD simulations with GROMACS 4.6.3 [37] and the CHARMM22/CMAP force field [38]. Each protein was soaked in a dodecahedral box of TIP3P [39] and 150 mM NaCl. After a step of energy minimization with the steepest descent algorithm (10000 iterations), the solvent was equilibrated in the NVT ensemble for 400 ps at 296 K, constraining the protein heavy atoms. We carried out 5 ns of pressurization and 9 ns of thermalization steps in the NPT and NVT ensemble, respectively. We collected four different simulations for the *wild-type* enzymes and two for the mutant variant initializing the system with different initial velocities. For each of them, we collected productive simulations of 500 ns each (350 ns for mutant simulation) in the NVT ensemble at 296 K and 1 bar, with a 2 fs time-step. We used the Particle-mesh Ewald (PME) switch summation scheme [40] for long-range electrostatic interactions and 0.9 and 0.8 nm switch cutoffs for Van der Waals and Coulomb interactions, respectively. The non-bonded pair list was updated every 10 steps and conformations were stored every 20 ps. We employed the LINCS algorithm [41] to constrain the heavy atom bond lengths, to use a 2 fs time step.

### Analysis of MD simulations

We calculated the main chain Root Mean Square Deviation (RMSD) using the starting structures as a reference to assess the stability of the trajectories and to rule out deterioration of the structure during the simulation time. We also calculated the time series of the protein radius of gyration and of the total, kinetic and potential energy. We verified that a stable coordination of Mg^2+^ and Cl^—^had been retained during the simulation time, monitoring the distances Mg^2+^-N127 and Cl^—^C44. We calculated the Cα Root Mean Square Fluctuation (RMSF) over non-overlapping windows of 10 ns, excluding the first ten ns, and then the profiles were averaged for each trajectory. Finally, we averaged the RMSF profiles from different replicates of the same system to obtain a single graph per enzyme.

### Principal Component Analysis (PCA)

We employed PCA-based approaches to evaluate the conformational sampling of the different trajectories and their overlap [42]. In particular, we calculated the Root Mean Square Inner Product (RMSIP) over the first ten principal components between each pair of independent simulations of the same system [43]. This value can range from 0 (when there is no correlation in the sampled phase space) to 1 (for completely overlapping simulations).

### Clustering-based Ensemble Similarity (CES) and Dimensionality-Reduction-based Ensemble Similarity (DRES)

We used CES and DRES as additional methods to evaluate the sampling achieved in our simulations. These methods have been designed to compute similarity measures between conformational ensembles and rely on the calculation of the Jensen-Shannon divergence between probability densities estimated from the ensembles under analyses [44]. CES and DRES differ in that they rely on different methods to estimate such probabilities. The CES method employs the Affinity Propagation (AP) clustering [45] method to partition the overall conformational space, and the probability distributions are estimated from the relative population of the ensembles in the conformations that populate the clusters.

The DRES methodology employs the Stochastic Proximity Embedding (SPE) [46] to represent the ensemble conformational space in a lower dimensional subspace of *d-*dimensions. The advantage of CES and DRES is that the comparison of probability densities not only provide an estimate of the similarity of different ensembles, but it also allow to evaluate if certain conformations occur with the same frequency in different simulations. The calculated values can span from 0 to log(2), where 0 value means that the two distributions are identical. We carried out these calculations with the Encore software [47] using a preference value of -10 for the CES analysis and a dimension of 6 for DRES analysis.

### Protein Structure Network (PSN) analysis

We used the PyInteraph suite of tools to compute the hydrogen-bonds, salt bridges and hydrophobic interactions in the MD ensembles [48]. The PSN method exploits graph theory to identify networks of interactions in a protein, defining the residues as nodes and the interactions as edges [49,50]. We used an estimate of the interaction persistence during the simulation time as edge weight, according to PyInteraph definition [48]. The criteria used to establish the existence of a link between two residues depend on the nature of the interaction: hydrophobic interactions, hydrogen bonds (H-bonds) or salt bridges. For the first class the center of mass of the residue is computed and an edge is created if the distance is below 0.65 nm to avoid to discard important contacts observed in the experimental 3D structure. To account for a H-bond, the donor and acceptor atoms have to be at a distance of 0.35 nm and the donor-hydrogen-acceptor angle greater than 120°. At last, for salt-bridges the distance between two charged groups belonging to different residues has to be lower than 0.45 nm.

## Results and discussion

### Multiple sequence alignment

**Fig 1:**
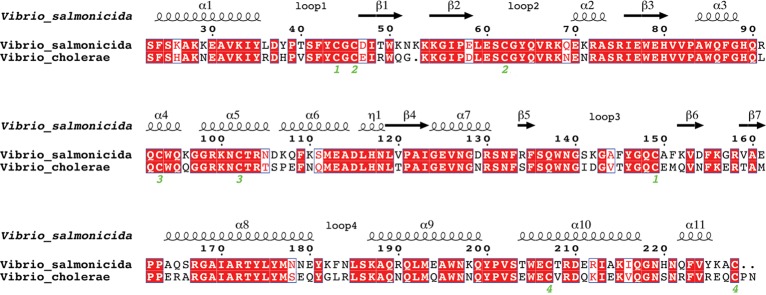
Sequence alignment of the homologs VsEndA (PDB: 2PU3) and VcEndA (PDB: 2G7F). The green numbers underline the disulfide bonds. The sequence numbering is based on *Vibrio vulnificus* endonuclease.

VsEndA and VcEndA are composed of 207 and 208 aminoacids respectively. The difference in sequence length is due to the insertion of a lysine in the psychrophilic variant, in position 52 (52A), and a proline plus an asparagine at the C-terminal of the mesophilic variant. In the sequence alignment between VsEndA and VcEndA, there are five regions where most of the amino acidic substitutions cluster: β1–2, α5–6, loop3 and β5–6 (the densest one), loop 4 and α10–11 (C-terminal) (Fig 1).

S1 Table displays the difference in amino acids composition of VsEndA with respect to VcEndA. The most evident difference is in the number of lysines, twelve more in VsEndA than in VcEndA, as already highlighted by Altermark et al. [24,25], a pattern suggested to be related either to adaptation to cold or to high salinity. In the psychrophilic enzyme the total number of negatively charged residues decreases, due to an increase in aspartic acids (+2) and a decrease in glutamic acids (-4). We observed a smaller arginine content in VsEndA (-2) in agreement with common traits observed for other cold-adapted enzymes. The polar residues are markedly lower in VsEndA, mainly due to a loss of asparagines (-4) and glutamines (-4), in favor of lysines. There is also a decrease in VsEndA in bulky hydrophobic residues (valine -2, methionine -2, leucine -1), while there is a gain in alanines (+4). Finally in VsEndA there are a higher number of aromatic residues (phenylalanine +3, tyrosine +2).

Most of the amino acidic substitutions are located throughout the surface of the protein with the exception of four buried inside the core: T120V (on β4, close to the catalytic H80), M151F (close to β5–6), A166S (on α8) and L183F (on loop 4, in the area below α3–6). This brings two more bulky aromatic residues in the hydrophobic core. Cold-adapted enzymes often feature a higher glycine content than their warm-adapted counterparts. This is not the case of VsEndA since we did not observe differences in the glycine number between the two endonucleases. Another common signature of cold-adapted enzymes is a low number of proline residues, which enhance structural rigidity and are often located in turns. In line with these findings, we observed a lower proline content in VsEndA (7 and 9 in VsEndA and VcEndA, respectively). In VcEndA the first proline (P107) is located in a turn between α5 and α6, and might increase local rigidity in this region. The second one (P229) is inserted at the C-terminal of VcEndA sequence and is not expected to have a major impact on overall thermostability and kinetic parameters.

It should be noted that the comparison of only two sequences can be misleading as many of the amino acidic differences can be the result of genetic drift rather than environmental adaptation. Thus we decided to proceed with a multiple alignment of VsEndA with homologous sequences (see Materials and Methods). The strategy pursued was to identify those amino acidic differences between VsEndA and other mesophilic variants which were also conserved in the sequences of EndA proteins isolated from other psychrophilic and psychrotolerant organisms (optimal temperature between 20 and 28°C and the ability to survive at 4°C and not at 37°C).

The closest homologs in terms of sequence identity (down to 70%) are from the genus *Vibrio*, and they are all marine or estuarine microorganisms, mostly halophiles and mesophiles [51]. The other most populated genus in the list belong to the following organisms: *Photobacterium*, *Cronobacter*, *Citrobacter*, *Klebsiella*, *Serratia*, *Shigella*, *Shewanella*, *Aeromonas* and *Pseudomonas* (with sequence identities from 68% to 54%). The list of the habitat and optimal growth temperatures are reported in S2 Table in the Supporting Information.

The analysis allowed us to discriminate among the amino acidic substitutions potentially related to cold adaptation and the non-relevant ones. We classified the different variants according to the optimal growth temperature found in the literature for each microorganism. It should be noted that the reported optimal growth temperature might not necessarily coincide with the actual temperature of growth. This leads to inherent uncertainties related to the existence of multiple subtypes of microorganisms with different optimal parameters. Further complications arise from the lack of standard routines used by different research groups to determine the temperature range and optima. The curation of the dataset to use for the alignment and its critical assessment is thus of crucial importance.

**Fig 2:**
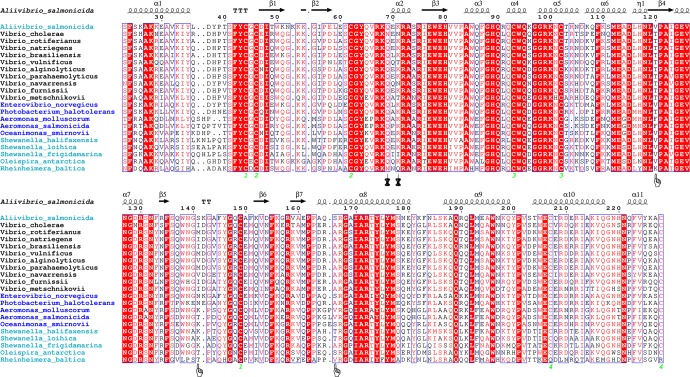
Multiple sequence alignment of psychrophilic, psychrotolerant and mesophilic homologs of VsEndA. The sequences of psychrophilic, psychrotolerant and mesophilic enzymes are highlighted in turquoise, blue and black, respectively. The hand icon marks the amino acidic substitutions selected in this study, whereas the pin marks those analyzed by Niiranen et al. [27]. The green numbers highlight the disulfide bridges. The sequences are ranked by sequence identity.

Fig 2 reports the multiple sequence alignment. The most relevant substitutions for cold adaptation are likely to be T120V, S141I and A166S. The first one is located on β4, which is part of the ββ-α motif, deeply inside the enzyme core and close to the catalytic H80 and the Mg^2+^ coordinating E79. The threonine is conserved in the mesophilic sequences and in *Photobacterium halotolerans* (psychrotolerant), while most of the psychrophiles possess a valine or a phenylalanine in this position. The solvent exposed S141I is located in loop 3 near the binding site, and in the other psychrophilic or psychrotolerant sequences it is either a polar asparagine/threonine or a charged lysine. The other *Vibrios* endonucleases present hydrophobic residues varying between isoleucine, methionine and valine. Finally, A166S is on α8 facing the enzyme core and only five cold-adapted organisms have serine or threonine at this position, whereas most of the other sequences feature a valine or alanine.

The amino acidic substitutions characterized by Niiranen et al. [27] (N69Q and N71K) were found also in other mesophilic *Vibrio* enzymes in our multiple sequence alignment, suggesting that these amino acids are unlikely to have a major impact on cold adaptation of VsEndA.

### Evaluation of the conformational sampling in MD simulations

**Fig 3:**
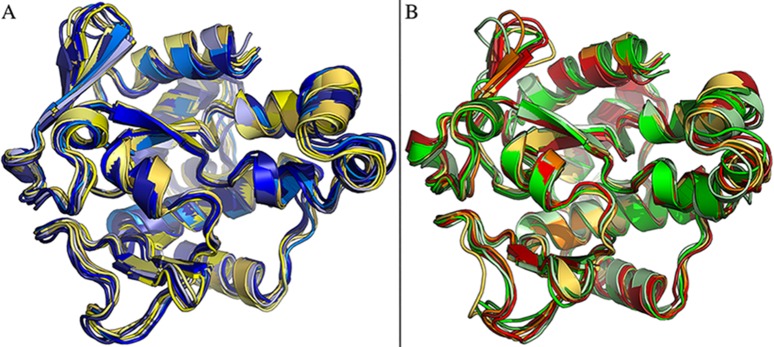
Snapshots from MD of VsEndA and VcEndA. We report seven different frames from MD replicates 3 and 1 of VsEndA (A) and VcEndA (B), respectively. On the left the aligned snapshots of VsEndA (A) and on the right of VcEndA (B). The frames have been selected to represent the major structural changes observed in the simulations.

To examine the specific role of the amino acids identified from sequence comparisons and to investigate the dynamical properties of the enzymes, four independent all-atom (MD) simulations in explicit solvent of 500 ns each were carried out for VsEndA and VcEndA (for a total of 2 μs for each homolog) (Fig 3).

Nowadays this trajectory time length is modest. Nevertheless, to our best knowledge, it has reached one of the longest sampling in MD studies of cold-adapted enzymes. We recently reviewed the computational studies in the field giving an overview of the analysis methods employed and simulation time span [52]. The first study of Brandsdal et al. [53] in 1999 on trypsin collected in total 1.2 ns of trajectories and in more recent years the simulation length reached higher sampling, such as in Martinez et al. on subtilisin S41 [54] with 50 ns. The longest trajectory was ran by Óskarsson et al. [55] in 2016 on cold-adapted subtilase for 600 ns.

Most of the MD replicates reach stable main-chain RMSD values after 10 ns of simulation, with few exceptions where more than 250 ns were required to reach a plateau (S2 Fig). Also other properties are rather stable during the simulation time, such as the radius of gyration (S3 Fig) and the energy profiles (S4 and S5 Figs).

We also monitored the coordination modes of the catalytic Mg^2+^ and the Cl^-^ to assess the stability of the trajectories and the quality of the sampled structures (S6 Fig). Indeed, these two ions are an integral part of the 3D architecture and their loss could cause major structural rearrangements.

**Fig 4:**
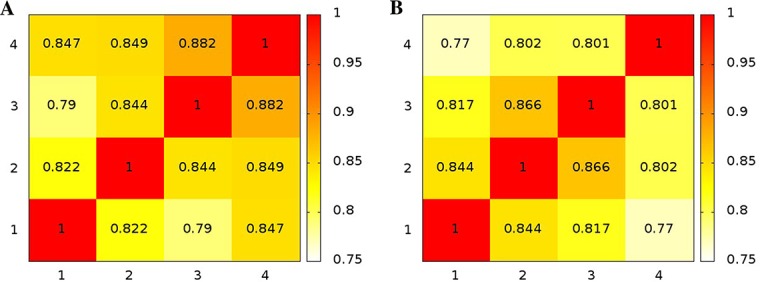
Similarity between the different ensembles measured by RMSIP. The heatmaps illustrate the RMSIP for intra-replicates comparisons of VsEndA (A) and VcEndA (B) MD simulations.

We calculated the RMSIP from PCA analysis for a proper assessment of the sampling during the simulations. We have computed the RMSIP between each replicate of the same system over the first ten components. Fig 4 shows that the RMSIP values are higher than 0.80 with few exceptions.

**Fig 5:**
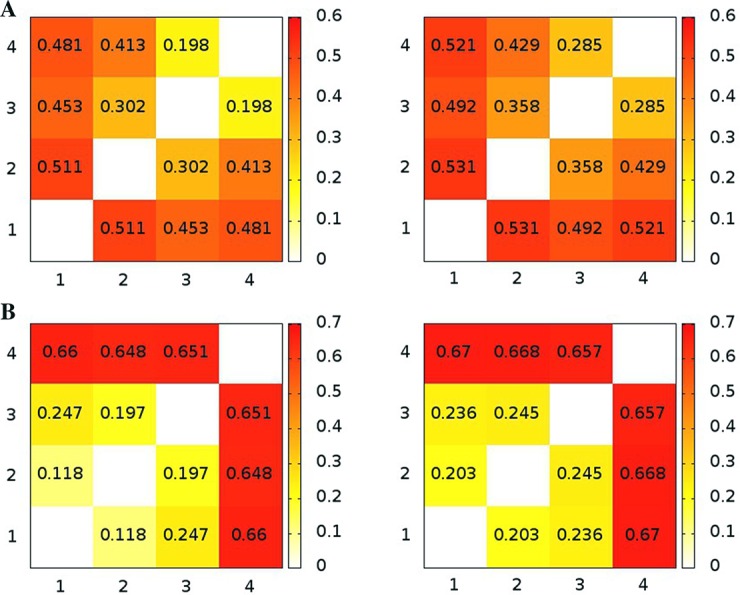
Similarity between the different ensembles measured by CES and DRES. The heatmaps illustrate the results achieved with the clustering-based method (CES, left) and the dimensionality reduction method (DRES, right) for intra-replicates comparisons of VsEndA (A) and VcEndA (B).

PCA can be often reductive in comparison between different MD simulations [56]. Thus, we also employed additional methods that allow to accurately estimate the probability distribution of different ensembles and compare them, namely: the clustering based ensemble similarity (CES) and dimensionality reduction based ensemble similarity (DRES) [44],[47]. CES and DRES scores close to zero mean that the two ensembles have very similar probability distributions, whereas values closer to 0.69 indicate that the two ensembles are non-overlapping. RMSIP, CES and DRES analyses show that replicate 1 in VsEndA and 4 in VcEndA are sampling different conformations with respect to the other replicates of the same system (Figs 4 and 5). We noticed that the differences in RMSIP values were less pronounced than the ones observed with CES and DRES methods. Thus, we discarded the two replicates mentioned above from further analysis. Indeed, we cannot conclude with such a limited number of conventional MD replicates if they are sampling statistically relevant conformations on the free energy surface.

### Flexibility profiles

**Fig 6:**
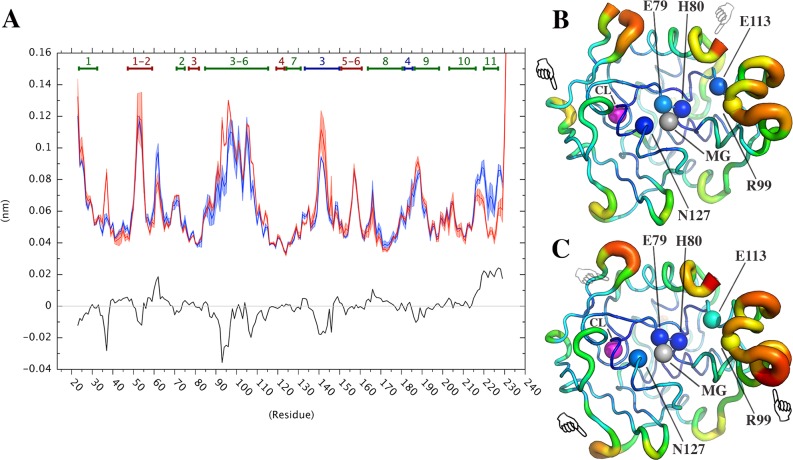
Flexibility profiles for VsEndA and VcEndA. (A) RMSF profiles for VsEndA (blue), VcEndA (red) and the difference between the first and the second (black). The shaded area above and below the single profiles represents the standard deviation. The profiles have been averaged over three replicates per enzyme. The RMSF of these single simulations have been averaged over windows of ten ns. On the right the 3D structures of VsEndA (B) and VcEndA (C) represented with the putty cartoon in PyMOL. The hand symbols indicate the region of different flexibility between the two enzymes. The spheres on the backbone place the positions of the catalytic residues.

To compare the flexibility of the two proteins, we used the Cα Root Mean Square Fluctuation (RMSF) (Fig 6A). Moreover, we used the average trace of the diagonalized covariance matrix (over three replicates for each system) as a metric to estimate the overall protein flexibility. We observed only a marginal difference in terms of overall flexibility between the mesophilic (1.27 ± 0.04 nm^2^) and psychrophilic enzyme (1.23 ± 0.27 nm^2^). Moreover, we observed only modest and very local changes in RMSF when we compared the RMSF profiles averaged over 10-ns time windows. The simulations of VsEndA reveal higher RMSF values for the conserved S59 and E60 located on a small helix turn, and at the C-terminal (from position 215 to 228). VcEndA also reveals a higher peak (0.03 nm) at the end of α1, for R36 (L36 in VsEndA) and the conserved E37, on α4 from residue 91 to 96 (highest value on Q92, 0.04 nm) and on loop3 on I142 (0.03 nm, a serine in VsEndA).

The multiple sequence alignment in Fig 2 shows that in position 36 the most represented residue is a glutamine, also in psychrophilic and psychrotolerant organisms, while the arginine is specific only in VcEndA. A leucine/alanine, as for VsEndA, is present also in four other sequences both mesophilic and psychrophilic. In the region 91–96 the only positions of variance are 91 and 96, but no similarity can be found between mesophilic or psychrophilic/psychrotolerant sequences. The only amino acidic substitution highlighted in the previous multiple alignment analysis is I141S, since all mesophilic sequences possess a hydrophobic residue (I/M/V). This finding is consistent with the structural alignment of the crystal structures of VsEndA, VcEndA and Vvn, showing that this is one of the regions interested by the major structural differences between the three homologs [24].

For both enzymes, excluding the N- and C- terminal, the most flexible secondary structures are those surrounding the active site, namely: β1–2, α3–6, loop3 and β5–6. The region comprising α8-loop4-α9 that is below α3–6, is moderately fluctuating (with a peak around 0.08 nm). From a mechanistic point of view α3–6, comprising about thirty amino acids, is of interest since it shows the highest RMSF values (together with the β1–2). Most importantly as shown in the crystal structure of Vvn [28] and in the QM/MM studies of Bueren-Calabuig et al. [29] (using normal mode analysis), it makes extensive contacts with the major groove of the DNA, anchoring the DNA strand. α6 also contains R99, which is important for DNA binding and catalysis. This residue interacts with the scissile phosphate on OP2 and it stabilizes the transition state charge over the cleaved bond [29]. Fig 6B and 6C show the proximity to the active site of the most flexible domains and that the core of the catalytic site is rigid for both enzymes.

### Protein Structure Network (PSN) calculations

Graph theory has been extensively employed in conjunction with MD to describe the structural organization of proteins. The study of the PSN can help in clarifying aspects related to protein function and stability, allosteric regulation, signal transduction or binding of a substrate [57–61]. Here, the PSN methodology was used to reveal the interaction network of the two enzymes along the trajectories and to underline the differences. We analyzed separately each type of interaction to reduce the high dimensional set of data and we focused our attention only on the different edges between the two enzymes and on those residues for which we found differences in the multiple sequence alignment or in the RMSF profiles.

Table 1 shows that VsEndA overall has more hydrophobic interactions and less electrostatics one than VcEndA. For both the interaction classes the difference in edges are spread all over the structures, both in the core and on the surface, and it is impossible to relate this variation to common cold-adapted trends such as lower core packing, lower surface polarity or higher surface hydrophobicity.

**Table 1:** Number of electrostatic and hydrophobic interactions for the two enzymes, calculated with Pyinteraph. In the polar+electrostatics section are excluded the interactions between the backbone atoms.

Enzyme	Hydrophobic	Polar + Electrostatics
**VsEndA**	21	26
**VcEndA**	16	48

**Fig 7:**
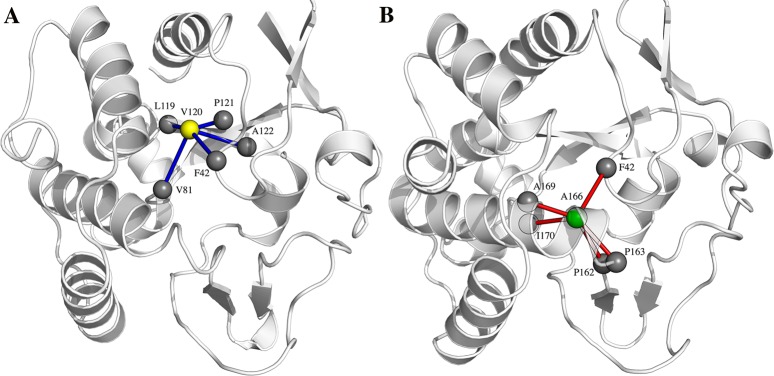
Different hydrophobic interaction clusters in VsEndA and VcEndA. The yellow and green colored spheres represent mutated residues between VsEndA (A) and VcEndA (B) respectively.

We also analyzed in details the clusters of each interaction type and the *hub* residues, i.e. those nodes that are connected by more than four edges. This topological features are generally key points for structure stability and signal transduction [60,62]. The PSN analyses of the two MD ensembles revealed two main clusters in the protein core that differ between the two enzymes (Fig 7). More in details, in VsEndA the amino acidic substitution T120V create a hub formed by 5 edges: F42 (loop1), V81 (α3), L119, P121, and A122 (β3). In VcEndA, on the contrary, S166A forms a hub with 5 edges: F42, P162 (which further connects with M161), P163, A169 and I170. With the exception of F42 the amino acids are located on α8 and a loop connecting to β5–6.

**Fig 8:**
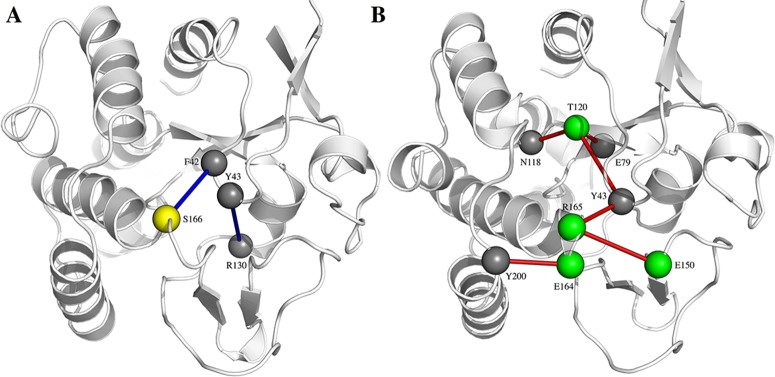
**Different electrostatic interactions in VsEndA (A) and VcEndA (B).** The yellow and green colored spheres represent mutated residues between VsEndA (A) and VcEndA (B) respectively.

**Fig 9:**
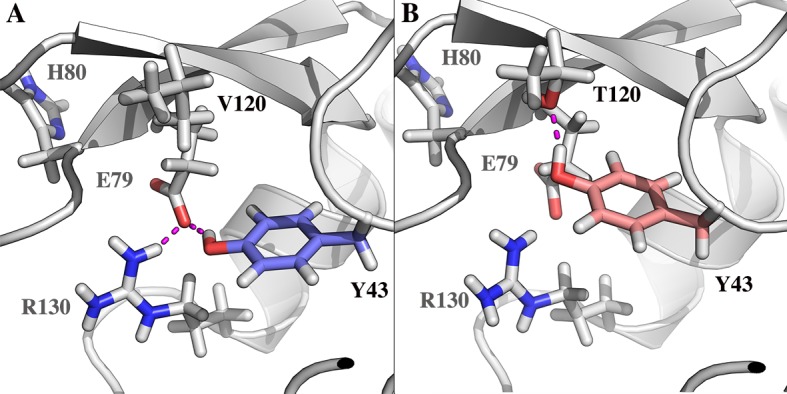
Interaction mode of Y43 with the ion coordinating residue E79. On the left the preferred conformation of VsEndA (A) and on the right by VcEndA (B).

The amino acidic substitution T120V proves to be important also regarding the electrostatic interaction networks. Indeed, the PSN analysis shows that the residue Y43 in VsEndA only interacts with the metal-binding residue E79 and its salt-bridge partner R130. In VcEndA the substitution from a valine to a threonine creates a second hydrogen-bond acceptor for Y43 side chain (Figs 8 and 9). In the mesophile, this tyrosine can populate two different conformational states depending on the interacting partner (T120 or E79). Due to its proximity to the catalytic residue H80 and the metal-biding residue E79, T120V becomes an interesting amino acidic substitution with a potential role in modulating the kinetic parameters.

The substitution A166S in VsEndA allows an interaction between S166 and the backbone of F42, whereas as previously stated A166 is involved in a hydrophobic cluster in VcEndA (Fig 8). Furthermore, the mesophile contains a salt bridge connecting α8 with loop3 and loop1: R165, E150 and Y43 (backbone). As seen from the multiple sequence alignment (Fig 2), this interaction is common in other mesophilic microorganisms, suggesting that this is a possible common warm-adapted feature.

In conclusion, the substitutions T120V and A166S, which were also highlighted in the multiple sequence alignment, are thus suitable candidates for experimental mutagenesis to assess their involvement in temperature adaptation of the enzyme.

**Fig 10:**
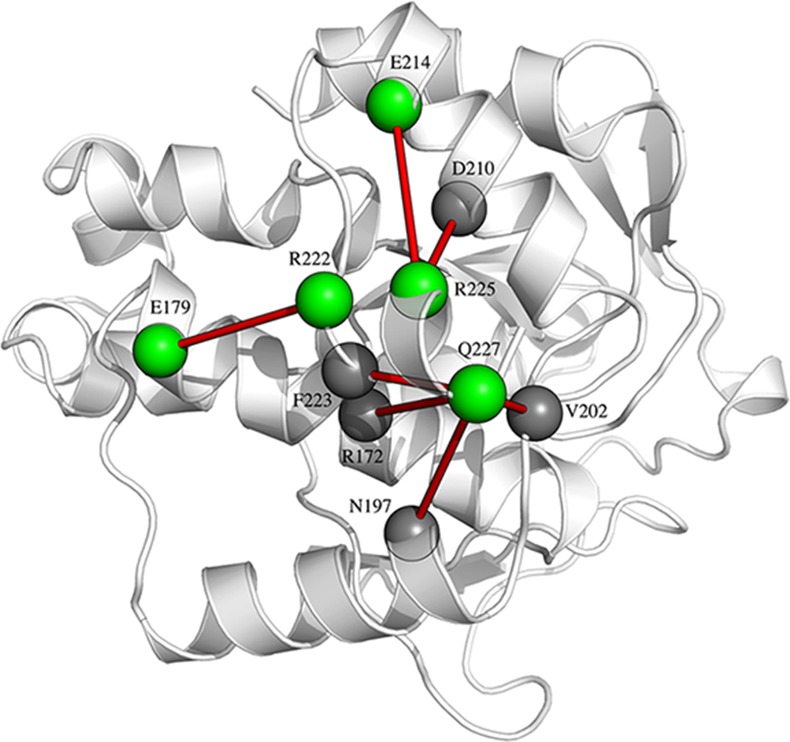
Clusters of interaction in the C-terminal of VcEndA. The green spheres represent the mutated residues with respect to VsEndA, while the dark grey the conserved one.

The C-terminal of VcEndA (α10–11) appears to be strongly stabilized by three electrostatic clusters: R225-D210-E214, R222-E179-E226 and Q227-R172-N197-V202-F223. The first and the latter anchor the domain to the main structure through interactions with α8 and α9 (Fig 10). These interactions are apparently weaker in VsEndA, as the stabilization and connection to the core of the enzyme becomes weaker. R222 and R225 are located at the center of the two salt-bridge clusters, which is a unique feature of VcEndA and not of the other homologs (Fig 2). These clusters might explain why the RMSF of the C-terminal is lower in the simulations of VcEndA compared to VsEndA.

**Fig 11:**
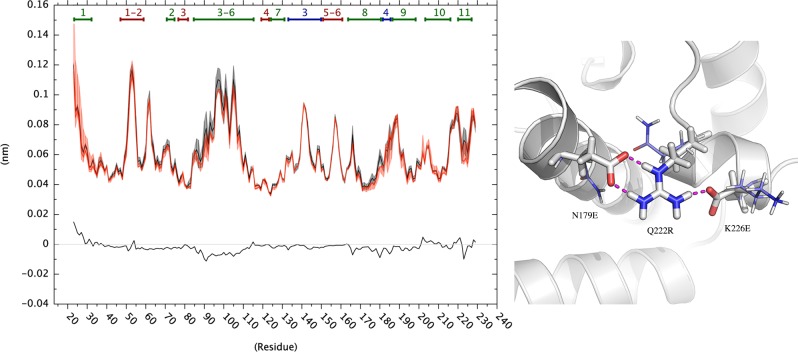
RMSF profiles for the simulations of VsEndA mutated in the C-terminal and Wild-type (WT). RMSF profiles for VsEndA WT (black), mutated (MUT, in red) and the difference between the first and the second (black). The shaded area above and below the single profiles represents the standard deviation. On the right are shown the mutated residues (in white), compared with the WT (pale blue).

To further investigate the role of these salt-bridges on the C-terminal dynamics of VcEndA, we modeled a mutant variant of VsEndA incorporating three amino acidic substitutions (N179E, Q222R and K226E) that allow to introduce the mesophilic salt-bridge cluster in the cold-adapted enzyme (S7–S10 Figs). We observed a decrease in RMSF of the C-terminal region of the VsEndA mutant variant with respect to the wild type cold-adapted enzyme (Fig 11), further supporting a role of the C-terminal electrostatic network in thermal stability.

## Conclusions

We collected in total two μs of trajectories for each enzyme and the ensemble similarity methods proved that three trajectories for each system were sampling overlapping regions of the conformational space. We employed multiple alignment, RMSF and PSN-MD analyses to characterize and compare the structure/dynamics of VsEndA and VcEndA, as well as to identify possible amino acidic substitutions related to temperature adaptation. Each method on its own is not able to give a complete picture, but their integration was the key to identify a subset of candidates for experimental mutagenesis. The multiple sequence alignment on a curated set of sequences allowed to discriminate amino acidic substitutions likely to be caused by genetic drift only and substitutions that can differentiate between mesophilic and psychrophilic/psychrotolerant endonucleases. The RMSF analyses allowed a description of protein mobility along the whole protein chain and to evaluate differences in local and overall flexibility. We did not observe marked differences in flexibility, in agreement with other recent studies on other systems [63,64]. This observation was not really surprising due to the close similarity of the 3D structures and sequences and the short timescales that we have been able to accurately assess. The only exception is the C-terminal region of the protein where a cluster of electrostatic interactions confers higher rigidity to the mesophilic variant. It is common idea that enhanced flexibility of regions close to the active site is one of the mechanisms related to adaptation to low temperature. We were not able to see such a trend in our analysis, where RMSF could be estimated only on the 10 ns timescales due to the averaging scheme employed.

Finally, the PSN calculations provided us with a detailed understanding of the differences underlying the interaction networks of the two enzymes. The amino acidic substitution T120V, located close to the catalytic residue H80 and the metal-coordinating residue E79, participate to a hydrophobic hub in the psychrophilic enzyme while in the mesophile it provides an alternative hydrogen-bond acceptor for Y43. No difference in the RMSF was found in this region, but we cannot rule out that changes can be observed on longer timescale. A166S introduces an H-bond between α8 and the loop region where is anchored Y43, possibly affecting the conformational state of the tyrosine.

## Supporting information

S1 Fig Structure of EndA. A) Secondary structural elements of VsEndA and VcEndA. B) The ββ-α motif is colored in red along with the Mg^2+^ coordinating residues. C) Surface representation of the EndA active site, with the bound DNA colored in purple, the Mg^2+^ in cyan and the ββ-α motif region in red.
(TIF)

S2 Fig Mainchain Root Mean Square Deviation (RMSD) for the simulations of VsEndA (A) and VcEndA (B).
(TIF)

S3 Fig Radius of gyration for the MD simulations of VsEndA (A) and VcEndA (B).
(TIF)

S4 Fig Energy profiles for the MD simulations of VsEndA. The potential energy (black, on the left), the kinetic energy (grey, in the middle) and the total energy (black, on the right) are shown for the MD replicates 1–4 of VsEndA. The unit of measure for the different energies is in kJ/mol.
(TIF)

S5 Fig Energy profiles for the MD simulations of VcEndA. The potential energy (black, on the left), the kinetic energy (grey, in the middle) and the total energy (black, on the right) are shown for the MD replicates 1–4 of VcEndA. The unit of measure for the different energies is in kJ/mol.
(TIF)

S6 Fig **Distance between the catalytic ion Mg**^**2+**^**, the structural Cl**^**-**^ **and their coordinating residues over the MD trajectories of VsEndA (A) and VcEndA (B).** The distance for Mg^2+^ on the left, while on the right Cl^-^. On the x-axis the unit of measure is ns, while on the y-axis it is nm.
(TIF)

S7 Fig Mainchain Root Mean Square Deviation (RMSD) for the MD simulations of VsEndA mutated in the C-terminal region. (TIF)

S8 Fig Radius of gyration for the MD simulations of VsEndA mutated in the C-terminal region. (TIF)

S9 Fig Distance between the catalytic ion Mg^2+^, the structural Cl^-^ and their coordinating residues over the MD trajectories of VsEndA mutated in the C-terminal region. The distances for Mg^2+^ and Cl^-^ ion are reported on the top and on the bottom, respectively.
(TIF)

S10 Fig Energetics profiles for the simulations of VsEndA mutated in the C-terminal. The potential energy (black, on the left), the kinetic energy (grey, in the middle) and the total energy (black, on the right) are shown for the MD replicates 1–2. The unit of measure for the different energies is in kJ/mol.
(TIF)

S1 Table Comparison of the aminoacidic content of VcEnda and VsEndA within the 52 substitution sites. The column on the right reported the net gain/loss per residue for VsEndA compared to VcEndA.
(DOCX)

S2 Table Classification of the microorganisms according to temperature optima and habitat. M stands for marine, B for brackish (organism that can grow with or without NaCl in the solvent) and NM for non-marine. ‘Opt T’ is the optimum of growth temperature and ‘T range’ indicates the lower and upper temperature limits for the microorganism. The question mark refers to optimal temperature not described in literature.
(DOCX)
