# Supplementary figures and images for: A comparative study of cold- and warm-adapted Endonucleases A using sequence analyses and molecular dynamics simulations
Source: PLoS One. 2017 Feb 13;12(2):e0169586. doi: 10.1371/journal.pone.0169586 (PMC5305256; doi:10.1371/journal.pone.0169586)

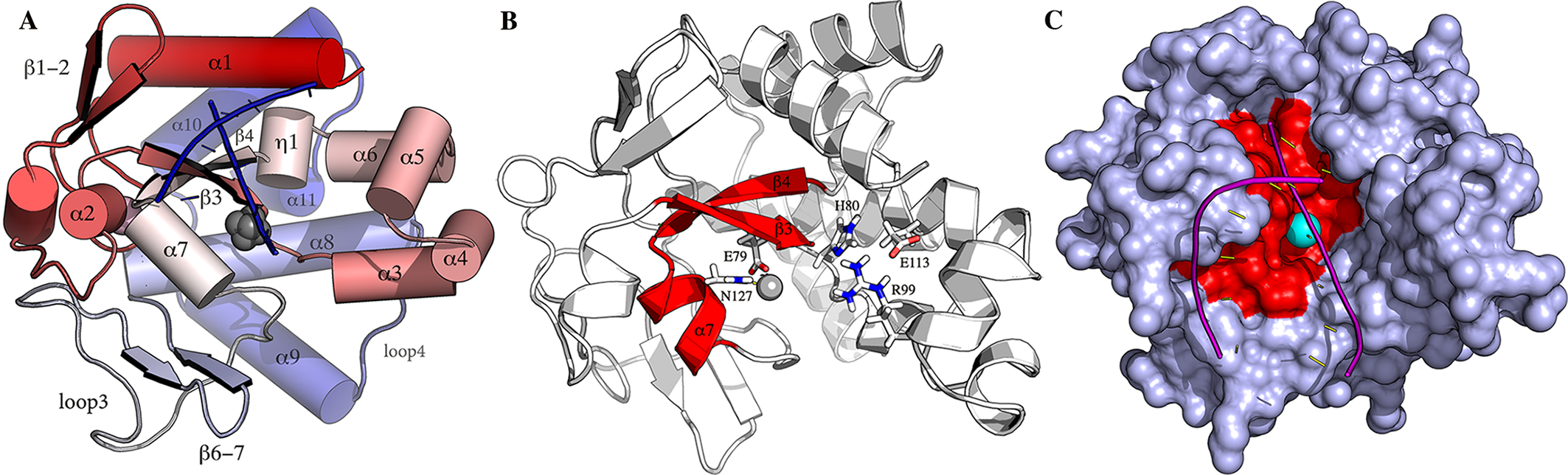

Supplement: S1 Fig — A) Secondary structural elements of VsEndA and VcEndA. B) The ββ-α motif is colored in red along with the Mg2+ coordinating residues. C) Surface representation of the EndA active site, with the bound DNA colored in purple, the Mg2+ in cyan and the ββ-α motif region in red. (TIF) [file pone.0169586.s001.tif]

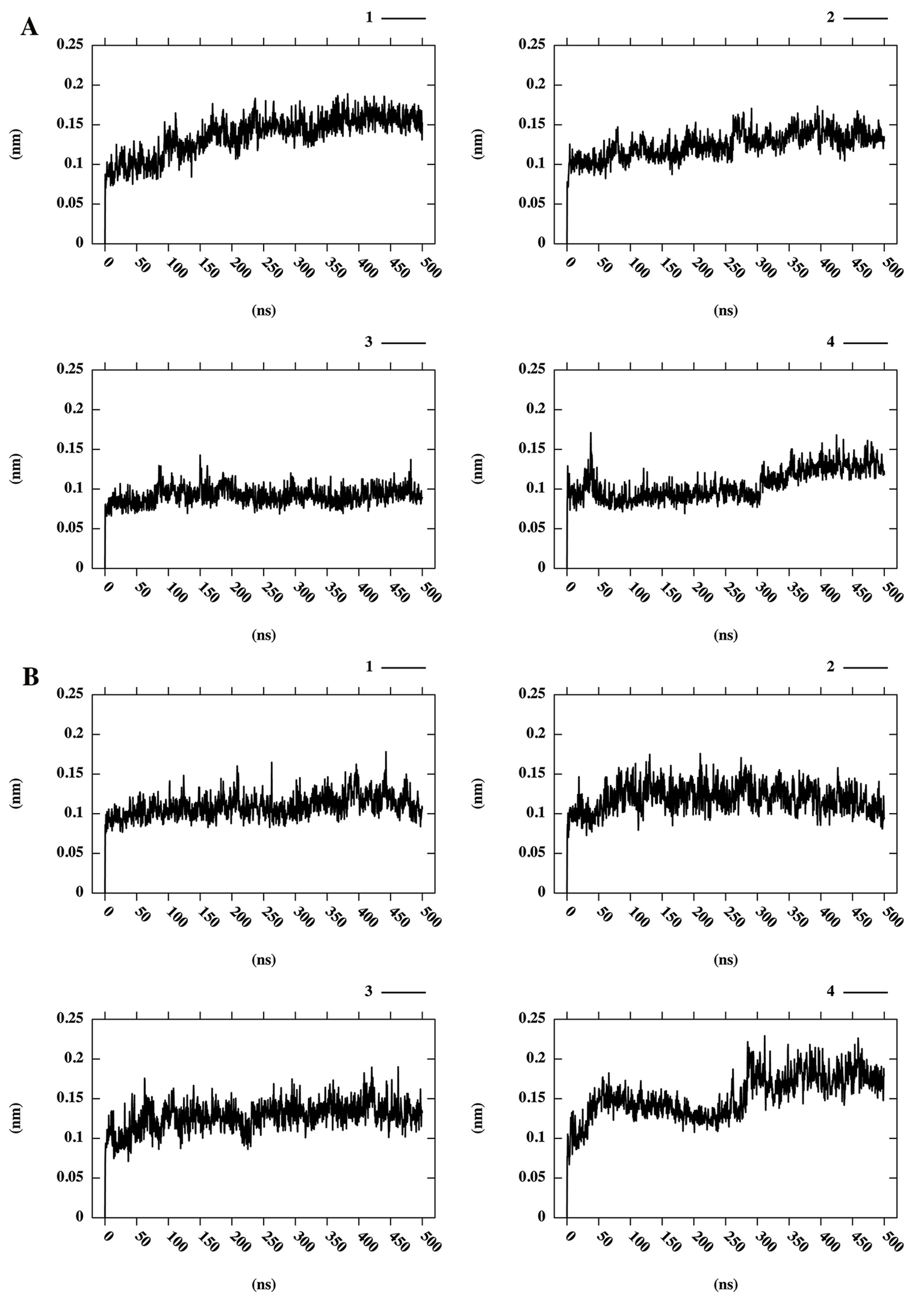

Supplement: S2 Fig — Mainchain Root Mean Square Deviation (RMSD) for the simulations of VsEndA (A) and VcEndA (B). (TIF) [file pone.0169586.s002.tif]

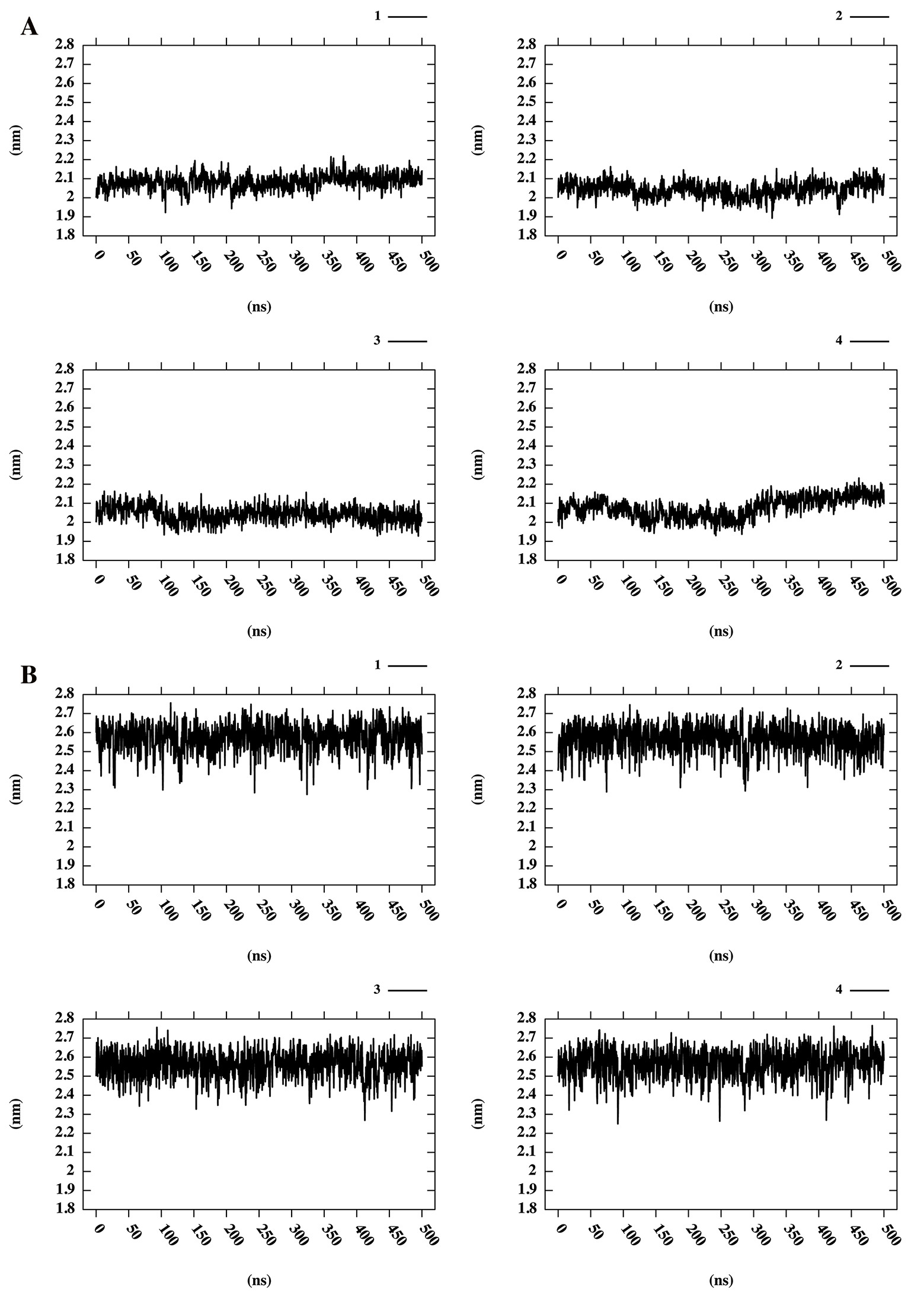

Supplement: S3 Fig — Radius of gyration for the MD simulations of VsEndA (A) and VcEndA (B). (TIF) [file pone.0169586.s003.tif]

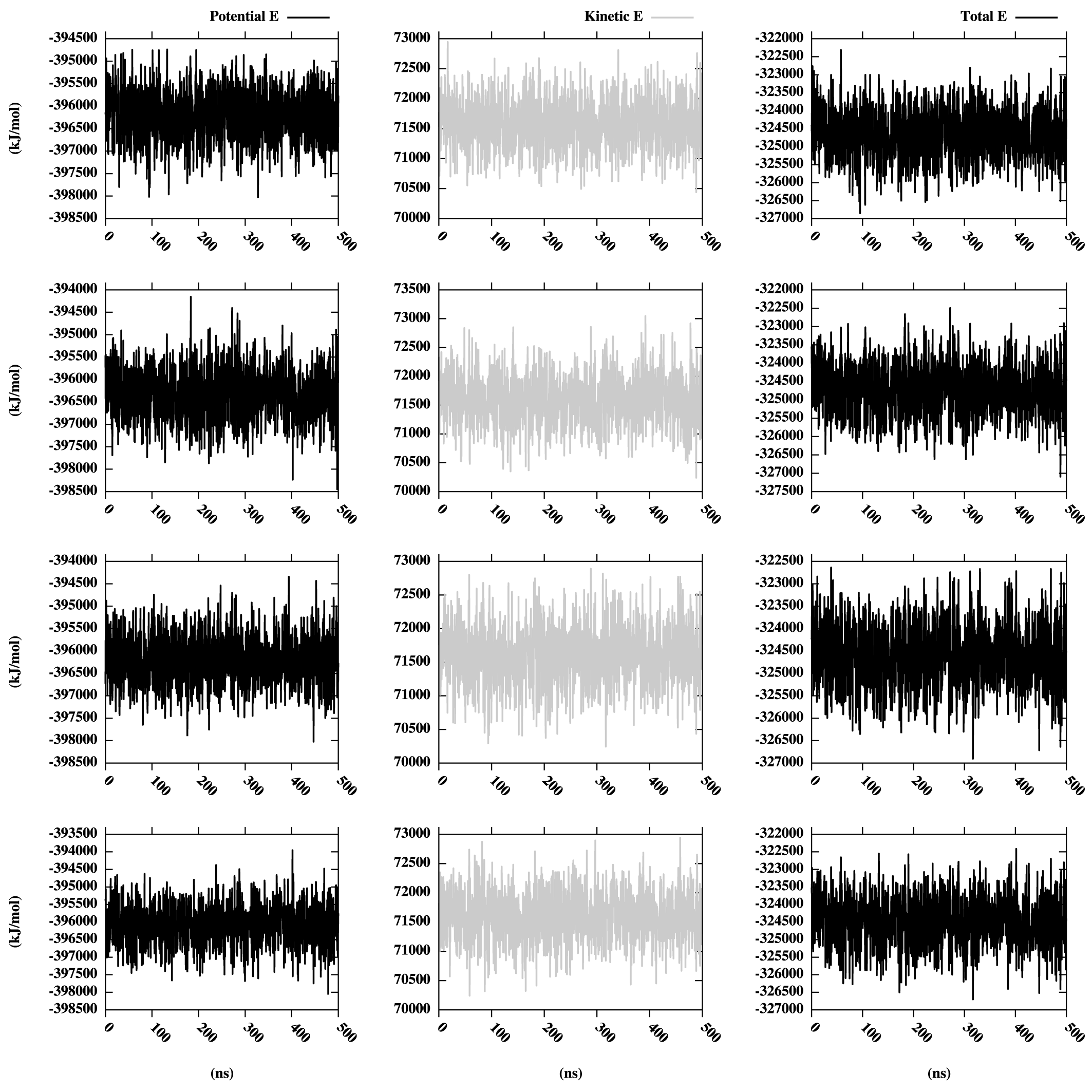

Supplement: S4 Fig — The potential energy (black, on the left), the kinetic energy (grey, in the middle) and the total energy (black, on the right) are shown for the MD replicates 1–4 of VsEndA. The unit of measure for the different energies is in kJ/mol. (TIF) [file pone.0169586.s004.tif]

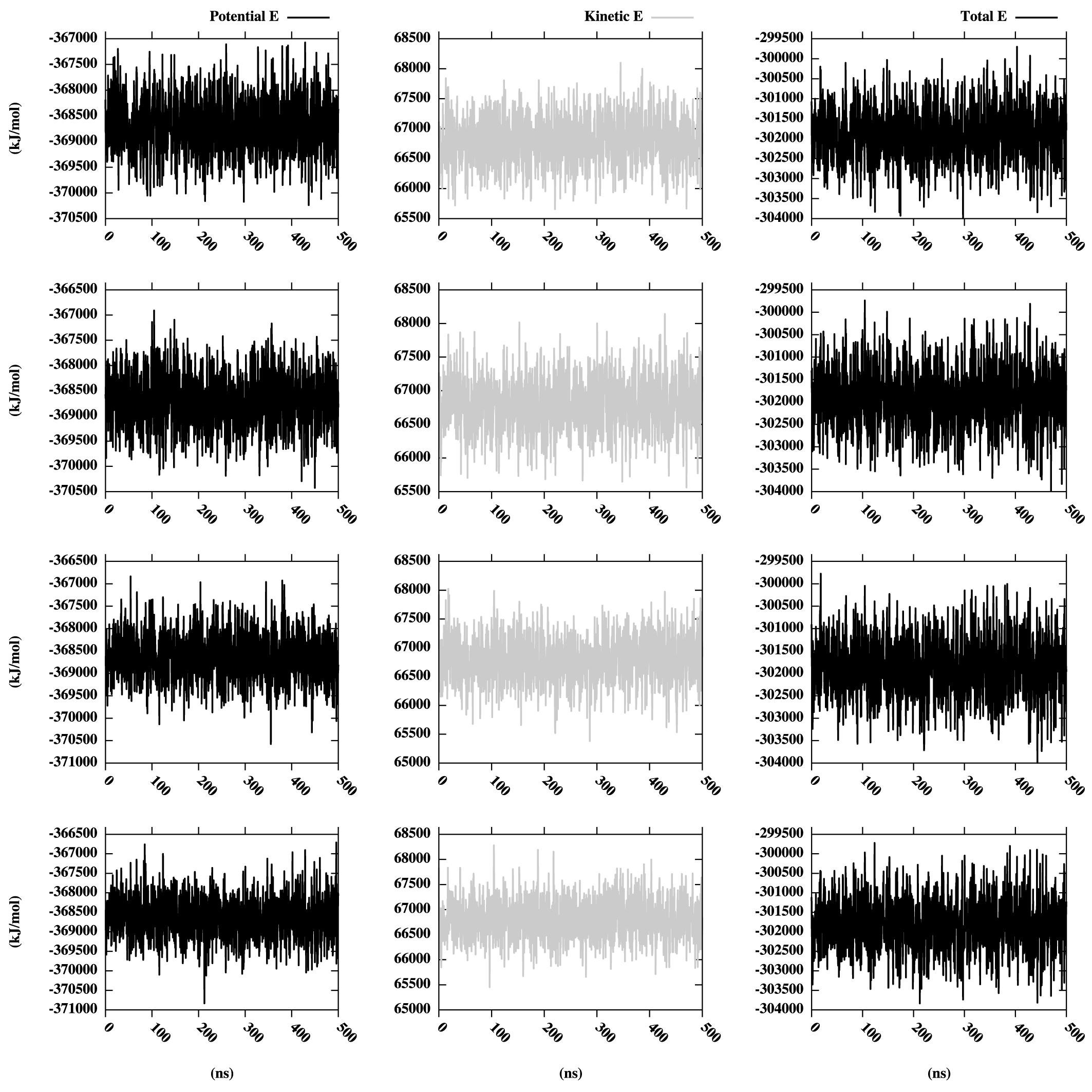

Supplement: S5 Fig — The potential energy (black, on the left), the kinetic energy (grey, in the middle) and the total energy (black, on the right) are shown for the MD replicates 1–4 of VcEndA. The unit of measure for the different energies is in kJ/mol. (TIF) [file pone.0169586.s005.tif]

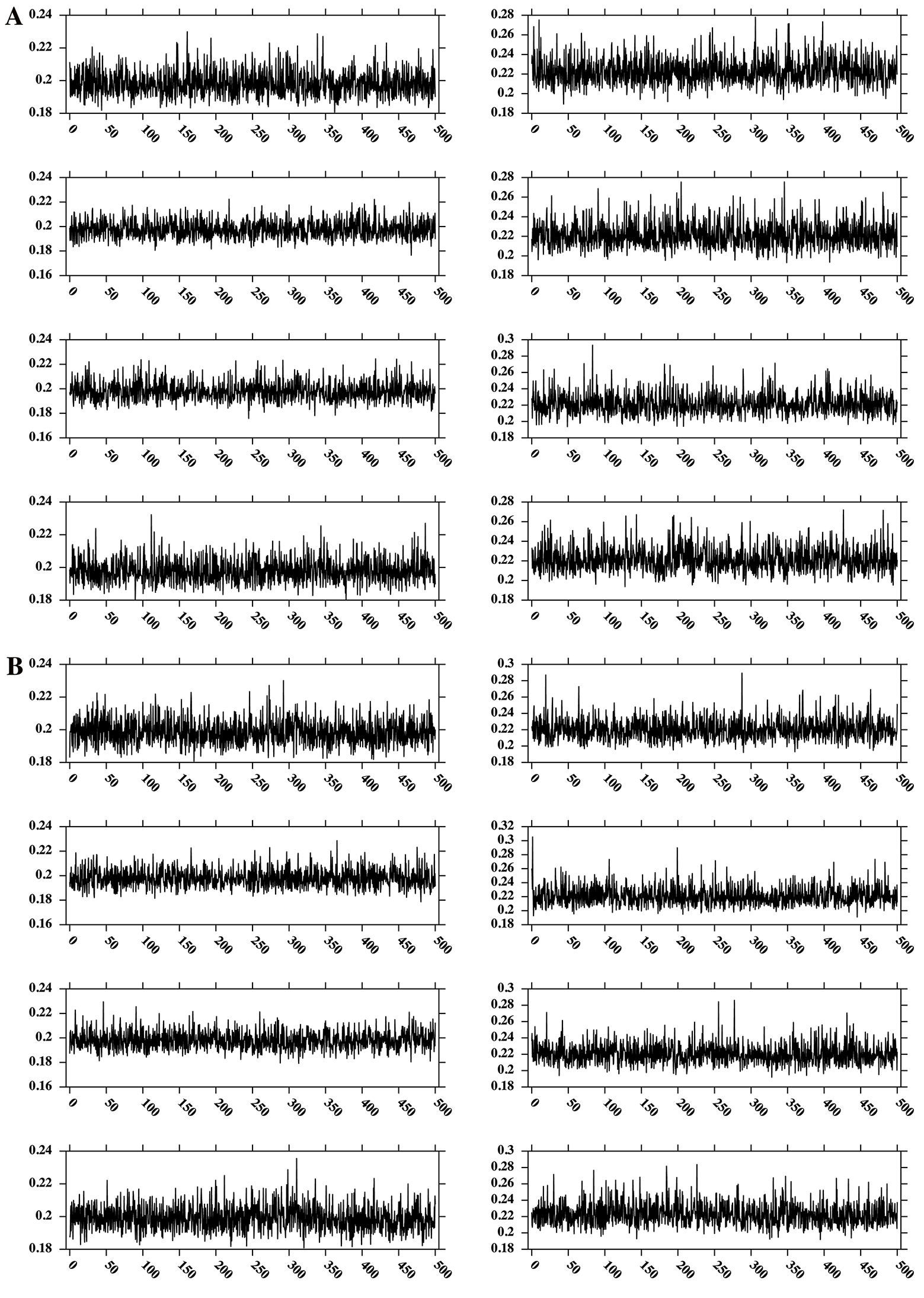

Supplement: S6 Fig — Distance between the catalytic ion Mg2+, the structural Cl- and their coordinating residues over the MD trajectories of VsEndA (A) and VcEndA (B). The distance for Mg2+ on the left, while on the right Cl-. On the x-axis the unit of measure is ns, while on the y-axis it is nm. (TIF) [file pone.0169586.s006.tif]

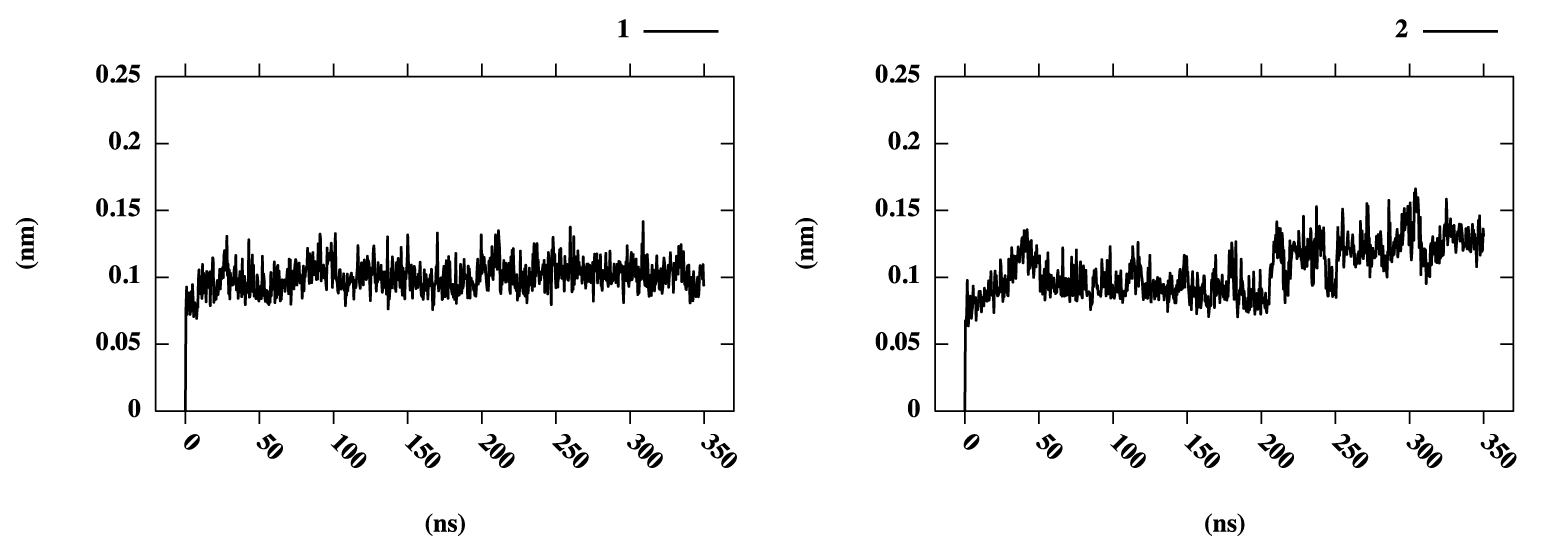

Supplement: S7 Fig — (TIF) [file pone.0169586.s007.tif]

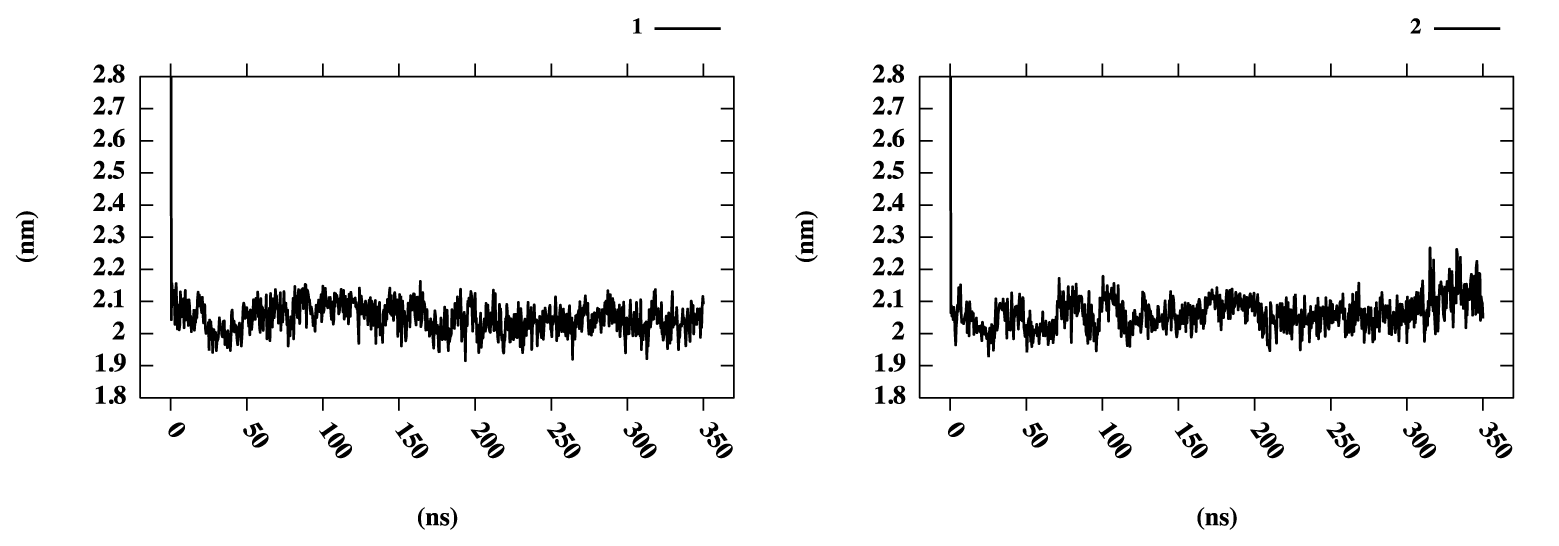

Supplement: S8 Fig — (TIF) [file pone.0169586.s008.tif]

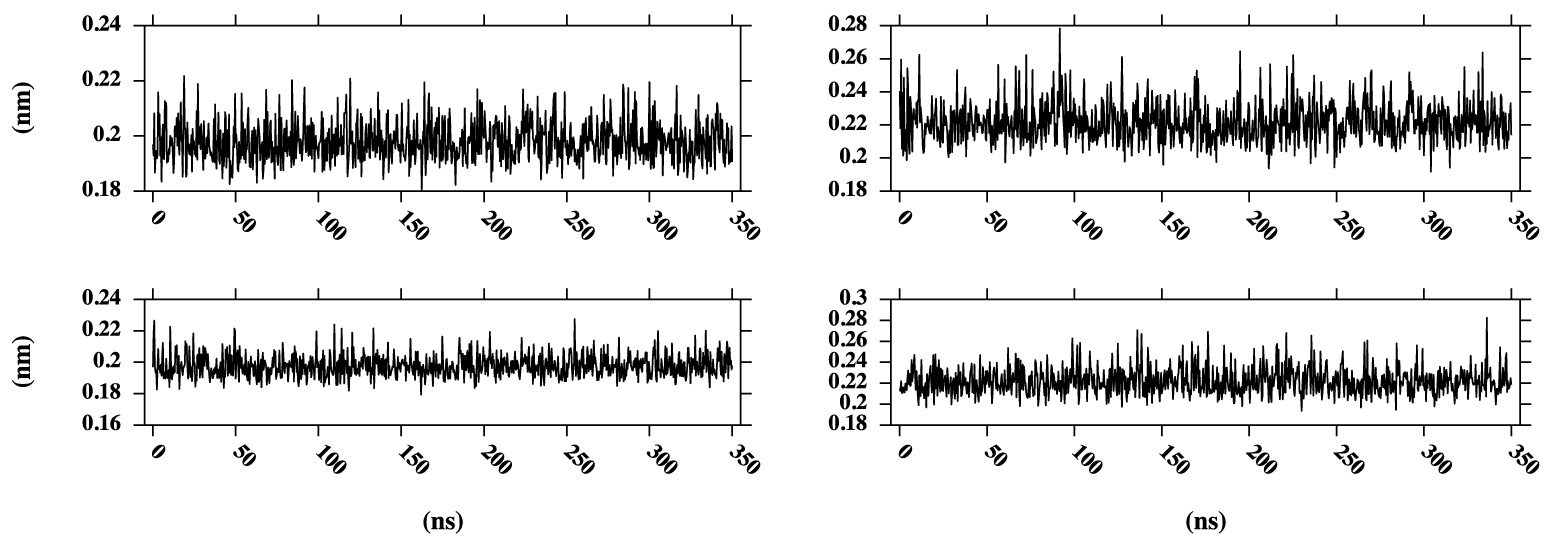

Supplement: S9 Fig — The distances for Mg2+ and Cl- ion are reported on the top and on the bottom, respectively. (TIF) [file pone.0169586.s009.tif]

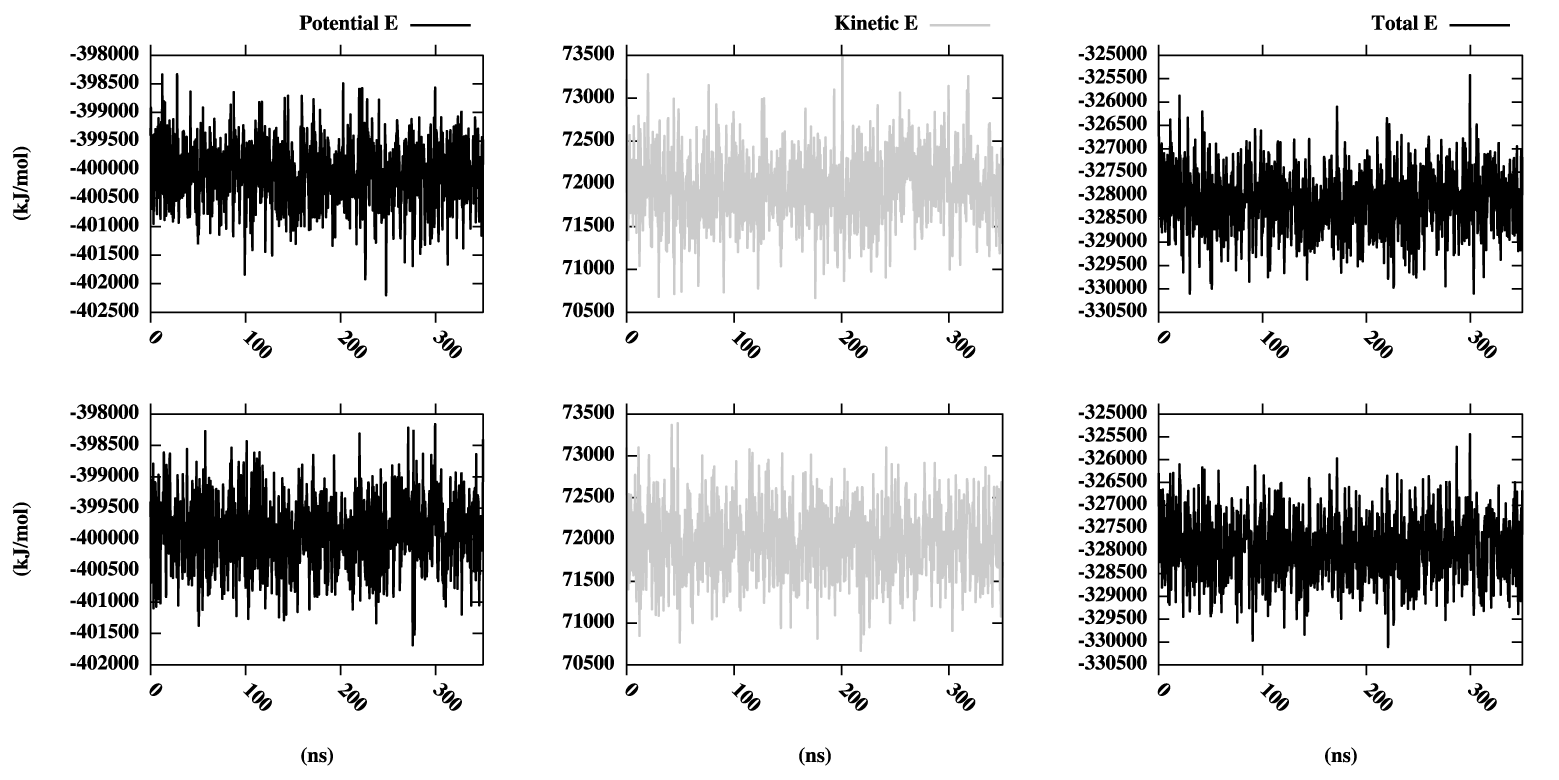

Supplement: S10 Fig — The potential energy (black, on the left), the kinetic energy (grey, in the middle) and the total energy (black, on the right) are shown for the MD replicates 1–2. The unit of measure for the different energies is in kJ/mol. (TIF) [file pone.0169586.s010.tif]
