# Supplementary material for: A comparative study of cold- and warm-adapted Endonucleases A using sequence analyses and molecular dynamics simulations
Source: PLoS One. 2017 Feb 13;12(2):e0169586. doi: 10.1371/journal.pone.0169586 (PMC5305256; doi:10.1371/journal.pone.0169586)
Supplement: S1 Table — The column on the right reported the net gain/loss per residue for VsEndA compared to VcEndA. (DOCX) [file pone.0169586.s011.docx]

| Residue | VcEndA | VsEndA |  |
| --- | --- | --- | --- |
| Lys (K) | 10 | 22 | 12 |
| Ala (A) | 12 | 16 | 4 |
| Phe (F) | 8 | 11 | 3 |
| Asp (D) | 5 | 7 | 2 |
| Tyr (Y) | 8 | 10 | 2 |
| Cys (C) | 8 | 8 | 0 |
| Ile (I) | 8 | 8 | 0 |
| Thr (T) | 6 | 6 | 0 |
| Trp (W) | 7 | 7 | 0 |
| Gly (G) | 15 | 14 | -1 |
| His (H) | 5 | 4 | -1 |
| Leu (L) | 8 | 7 | -1 |
| Ser (S) | 13 | 12 | -1 |
| Arg (R) | 16 | 14 | -2 |
| Met (M) | 5 | 3 | -2 |
| Pro (P) | 9 | 7 | -2 |
| Val (V) | 12 | 10 | -2 |
| Asn (N) | 17 | 13 | -4 |
| Gln (Q) | 19 | 15 | -4 |
| Glu (E) | 17 | 13 | -4 |
