# Supplementary material for: A comparative study of cold- and warm-adapted Endonucleases A using sequence analyses and molecular dynamics simulations
Source: PLoS One. 2017 Feb 13;12(2):e0169586. doi: 10.1371/journal.pone.0169586 (PMC5305256; doi:10.1371/journal.pone.0169586)
Supplement: S2 Table — M stands for marine, B for brackish (organism that can grow with or without NaCl in the solvent) and NM for non-marine. ‘Opt T’ is the optimum of growth temperature and ‘T range’ indicates the lower and upper temperature limits for the microorganism. The question mark refers to optimal temperature not described in literature. (DOCX) [file pone.0169586.s012.docx]

| **Microorganism** | **Opt T (˚C), (T range)** | **Environment** |
| --- | --- | --- |
| *Aliivibrio_salmonicida* | 15, 4 - 15 | M |
| *Vibrio_cholerae* | 37, 30 - 40 | B |
| *Vibrio_rotiferianus* | 28 – 40 | M |
| *Vibrio_natriegens* | 37, 30 - 40 | M |
| *Vibrio_brasiliensis* | 30 - 35 (no 4 or 40) | M |
| *Vibrio_vulnificus* | 37, 30 - 40 | M |
| *Vibrio_alginolyticus* | ? , 30 - 40 | M |
| *Vibrio_parahaemolyticus* | ? , 30 - 40 | M |
| *Vibrio_navarrensis* | ? , 10 - 40 | M |
| *Vibrio_furnissii* | ?, 30 - 40 | M |
| *Vibrio_metschnikovii* | ?, 30 - 40 | M |
| *Enterovibrio_norvegicus* | ?, 20 - 30 | M |
| *Photobacterium_halotolerans* | 28, 4 - 35 | M |
| *Oceanimonas_smirnovii* | 25 - 28, 10 - 45 | M |
| *Oleispira_antarctica* | 1-15, 8 - 25 | M |
| *Aeromonas_piscicola* | 25 - 30, 4 - 37 | M |
| *Aeromonas_molluscorum* | 25 - 30, 4 - 37 | M |
| *Shewanella_frigidamarina* | 21, 0 - 30 | M |
| *Shewanella_halifaxensis* | 10, 4 - 25 | M |
| *Shewanella_loihica* | 18, 0 - 42 | M |
| *Aeromonas_salmonicida* | 22 - 25, 2 - 30 | M |
| *Ferrimonas_balearica* | 25 - 30, 15 - 37 | M |
| *Rheinheimera_baltica* | 20 - 25, 4 - 30 | M |
